# Neurodegeneration, Neurogenesis, and Oxidative Stress 2015

**DOI:** 10.1155/2016/7632025

**Published:** 2016-02-02

**Authors:** Renata Santos, Anne-Laure Bulteau, Cláudio M. Gomes

**Affiliations:** ^1^Development of the Nervous System, IBENS, Ecole Normale Supérieure, 46 rue d'Ulm, 75230 Paris Cedex 05, France; ^2^Iprem University of Pau, 2 rue du Président Angot, 64000 Pau, France; ^3^Faculdade de Ciências, Universidade de Lisboa, Biosystems and Integrative Sciences Institute and Department of Chemistry and Biochemistry, Universidade de Lisboa, Campo Grande, 1749-016 Lisboa, Portugal

Oxidative stress is implicated in the pathophysiology of a wide variety of neurodegenerative disorders and its role in neurogenesis is becoming increasingly acknowledged. This special issue includes 8 articles that emphasize the implications of oxidative stress in neurodegeneration, neurotoxicity, and neurogenesis.

Several original and review articles discuss the role of oxidative stress in neurodegenerative disorders and upon brain injury. S. K. Singh et al. open the issue by presenting a comprehensive review on the pathology of Alzheimer's disease (AD). Two other review articles focus on specific aspects implicated in AD. L. Zuo et al. discuss how oxidative stress is related to AD progression and to the formation of A*β* plaques and tau neurofibrillary tangles. In addition, the authors examine evidence of epigenetic regulation of A*β* plaque formation in AD neurons and discuss different potential therapeutic approaches. J. S. Cristovão et al. concentrate on the role of metal ions in AD and overview different proteins implicated in AD whose metal binding properties may underlie important biochemical and regulatory processes occurring in the brain during the pathophysiological process. Hyperphosphorylation and aggregation of tau in neurons not only are a central feature in AD but are present in other neurodegenerative diseases, termed tauopathies. S. M. A. Naini and N. Soussi-Yanicostas review the relationship between tau pathology and oxidative stress and present arguments in favor of the hypothesis that these are key components of a pathologic vicious circle in tauopathies. In a different direction, X. Hu et al. summarize recent literature describing the contribution of oxidative stress to brain damage after intracerebral hemorrhage. H. J. Olguín et al. describe dopamine dysfunction as a consequence of oxidative stress and discuss its implication in disease conditions, such as in Parkinson's disease. In an original article, A. Seguin et al. present interesting data on a drug screen performed using two different models of Friedreich's ataxia, yeast and* Drosophila*. In the original article by B. P. Carreira et al., the authors examine the role of nitric oxide in neurogenesis in adult hippocampus following seizures. They show that although nitric oxide is beneficial in the early stages of production of newborn neural cells, it is detrimental to the survival of newly differentiated neurons due to inflammation.

